# Adhesive capsulitis of the shoulder, treatment with corticosteroid, corticosteroid with distension or treatment-as-usual; a randomised controlled trial in primary care

**DOI:** 10.1186/s12891-016-1081-0

**Published:** 2016-05-26

**Authors:** Satya Pal Sharma, Anders Bærheim, Rolf Moe-Nilssen, Alice Kvåle

**Affiliations:** Research Group, Section for General Practice, Department of Global Health and Primary Care, University of Bergen, Kalfarveien 31, N-5018 Bergen, Norway; Physiotherapy Research Group, Department of Global Public Health and Primary Care, University of Bergen, Bergen, Norway; Department of Occupational Therapy, Physiotherapy and Radiography, Bergen University College, Bergen, Norway

**Keywords:** Adhesive capsulitis, Corticosteroid, Distension, Frozen shoulder

## Abstract

**Background:**

Optimal management for adhesive shoulder capsulitis (frozen shoulder) is currently unclear. We intended to explore whether treatment by intra-articular injections with corticosteroid and distension is more effective than treating with corticosteroids alone or treatment-as-usual in a primary care setting in Norway.

**Methods:**

In this prospective randomised intention to treat parallel study, 106 patients were block randomised to three groups; 36 (analysed 35) receiving steroid injection and Lidocaine (IS), 34 receiving steroid and additional saline as distension (ISD) and 36 had treatment-as-usual (TAU). Intervention groups received four injections within 8 weeks, assessed on 1st visit, at the 4th and 8th week. Outcomes were Shoulder Pain and Disability Index (SPADI), Numerical pain rating scale (NPRS) and passive range of motion (PROM). Postal assessment was repeated after 1 year for SPADI. Patients in the IS and ISD groups were “blinded” for intervention received and the assessor was “blinded” to group allocation.

**Results:**

At baseline there were no differences between groups in outcome measures. There were no statistical significant differences between the intervention groups in SPADI, NPRS and PROM at baseline, at short-term (4-and 8 weeks) or long-term (12 months). There were statistically significant differences (*p* < 0.01) in change scores at short-term for SPADI when comparing the IS and TAU groups (-20.8; CI-28.9 to -12.7), and the ISD and TAU groups (-21.7; CI-29.4 to -14.0), respectively for NPRS (-2.0; CI-2.8 to -1.1 and -2.2; CI-3.0 to -1.4), and for PROM, but not at long-term for SPADI (*p* > 0.05).

Effect size (ES) at 8 weeks was large between both injection groups and TAU (ES 1.2). At 12 months ES was reduced to 0.3 and 0.4 respectively. Transitory side effects as flushing and after-pain were reported by 14 % in intervention groups.

**Conclusion:**

This intention to treat RCT in primary care indicates that four injections with corticosteroid with or without distension, given with increasing intervals during 8 weeks, were better than treatment-as-usual in treatment of adhesive shoulder capsulitis. However, in the long run no difference was found between any of the groups, indicating that natural healing takes place independent of treatment or not.

**Trial registration:**

ClinicalTrials.gov, https://clinicaltrials.gov/ identifier: NCT01570985

## Background

Adhesive capsulitis of the shoulder, also called frozen shoulder, has a prevalence of 2 to 5 % of the general population, but among diabetic patients the prevalence ranged from 11 to 30 % [1, 2]. There is a strong correlation between adhesive capsulitis and other medical conditions such as diabetes, rheumatic disease, heart disease, hyperthyreosis [3]. Adhesive capsulitis occurs mostly in middle age [4–6] and women between 50 and 60 years are most commonly affected [7]. Both shoulders can be affected simultaneously and/or the other side can be affected a few years later [7, 8]. Shoulder stiffness and pain interferes considerably with activities of daily living, and may be associated with increased sick leave in people of working age and incapacity in the elderly.

Adhesive capsulitis is a long-lasting disorder with spontaneous onset of pain and progressive stiffness [9]. It generally involves reduced movement of the gleno-humeral joint in several planes, with most restriction of external rotation, some restriction of abduction and least affection of internal rotation carried out passively, also called the capsular pattern [5, 6]. Adhesive capsulitis is primarily a clinical diagnosis and radiography can be complementary in the diagnosis [10, 11]. Pathophysiologically, thickening and contracture of the inferior capsule [12], contracture of the rotator interval, coraco-humeral ligament and anterior capsule with a combination of synovial inflammation and capsular fibrosis, has been described [10]. Bunker et al. found the histo-pathological picture comparable to Dupuytren’s disease of the hand with no inflammation and no synovial involvement [13]. The natural history remains controversial. Earlier studies considered the condition as self-limiting, lasting for 2 to 3 years, reporting that the majority of patients would get almost complete recovery or full recovery [14, 15]. Other authors report long-term pain and stiffness for several years [16–18]. For convenience, the condition is divided into three phases; the painful phase lasting from 3 to 9 months, followed by a freezing phase with progressive stiffness lasting from 4 to 12 months and finally, the recovery phase with gradual return of movement, lasting 5–26 months [19, 20]. Some have divided the condition into four stages, based on the correlation of findings on physical examination and arthroscopic examination [21].

Commonly used conservative therapies for adhesive capsulitis include non-steroidal anti-inflammatory drugs, intra-articular glucocorticosteroid injections, oral gluco-corticosteroid medication, physical therapy, manipulation under anaesthesia and hydrodilatation [22]. However, despite the amount of research in the topic, results still appear to be inconclusive regarding effectiveness of the different treatment modalities [23, 24]. In hydrodilatation or arthrographic distension procedures, an intra-articular injection is performed under fluoroscopy with local anaesthetics, normal saline and often with contrast medium. Most of the interventional studies with corticosteroid injections, with or without hydrodilatation (distension), have been done with single corticosteroid injection under fluoroscopy or ultrasound guided, either sub-acromial or intra-articular or both. Van der Windt et al. [25] used up to a maximum of three intra-articular injections over 6 weeks. According to Cyriax’s treatment method [1], adhesive capsulitis is often treated with between three to six corticosteroid intra-articular injections with increasing interval between injections, which is also supported by others [4–6, 26]. A short term efficacy of arthrographic distension with normal saline and corticosteroid versus placebo was demonstrated in a randomised controlled trial (RCT) in patients with painful stiff shoulder [27]. A systematic Cochrane review regarding efficacy of hydrodilatation concludes: “there is “silver” level evidence that arthrographic distension with saline and steroid provides short-term benefits in pain, range of movement and function in adhesive capsulitis. It is uncertain whether this is better than alternative interventions” [28]. Hydrodilatation studies [29–31] did not demonstrate any statistically significant differences in functional outcome compared to steroid injection [32].

The present study has followed the existing practice of treating patients with adhesive capsulitis in primary care in Norway. In a pilot trial, there was no clinically significant difference in overall results between corticosteroid alone and corticosteroid with distension [33]. The aim of this study was to elucidate the effect, if any, of multiple corticosteroid injections with distension as compared to multiple corticosteroid injections alone, to treatment-as-usual.

## Methods

This RCT comprises two parallel intervention groups and a control group allocating equal number of patients. The intervention period lasted 8 weeks, with a postal follow-up after 1 year. The patients were recruited from the city of Bergen and neighboring municipalities by referral from primary care (PC) practitioners from January 2010 to October 2013.

Included patients had to be above 18 years of age, should be able to understand and speak Norwegian, and have no contraindication for use of corticosteroids. Patients should have reduced passive range of motion (PROM) with a reduction of more than 30 % of two of three shoulder movements and none of the three movements (Abduction = ABD, External rotation = ER and Internal rotation = IR) should be normal. Patients with diabetes, asthma, pregnant women and breast feeding mothers were excluded from the study. Female patients in fertile age were asked about prevention.

Eligible patients were invited to participate in the study were randomly assigned to one of three groups according to serial no. on the closed envelope by one of authors (SPS). The block randomisation, using a block size of three, was carried out by one of the supervisors (AB). Possible permutations were strung together using a random cipher table. The resulting information on treatment was printed out and put in a closed envelope with the patient serial number outside. The envelope was to be opened after the inclusion of the patient. Treatment allocation was thereby “blinded” for both researcher and patient at the point of inclusion. The patients in the active intervention groups were not informed which treatment option (with or without distension) was carried out.

### Intervention

Intra-articular injections were administered by landmarks using posterior approach thus preventing the patients from seeing the size of syringe used. This was to avoid possible bias as the patients might consider treatment with distension and corticosteroid to be superior to corticosteroid alone. The injections were administered by one of the authors (SPS) who is both a general practitioner and a physiotherapist at a primary care center in municipality of Bergen and has several years of experience in treating adhesive capsulitis by intra-articular injections both by landmarks and ultrasound guided.

Patients in the steroid alone group (IS) received Triamcinolone 20 mg injection, with Lidocaine 10 mg/ml 3 ml and a total of 4 ml solution. Those in the distension group (ISD) also received steroid and Lidocaine (Triamcinolone 20 mg, 3 ml Lidocaine), but with additional physiological Sodium chloride 9 mg/ml, comprising a total volume from 8 ml and upwards to 20 ml. Limiting factors for injected volume were difficulty in further injection and/or increasing pain during injection. Injection to IS and ISD groups were given after inclusion on day 1, after 7, 17, and 31 days from the start. Adherence to planned intervention was assessed continuously by one of the authors (SPS). Patients receiving treatment-as-usual (TAU) were informed about the possibilities of optional conservative treatment, such as physiotherapy or pain medication other than corticosteroid injections or per oral corticosteroid medication until 61 days after inclusion.

### Outcome measures

The primary outcome was the Shoulder pain and disability index (SPADI), which measures a combination of pain and functional disability on a score from 0 to 100, a high score indicating more pain and disability [34]. The second outcome measure was pain intensity on average for the previous 7 days, measured on a 10-point Numerical pain rating scale (NPRS), where 0 meant no pain and 10 meant unbearable pain. PROM was measured in sideways elevation (abduction), internal rotation (by “Hand behind back” method) and external rotation. A plurimeter, found to be a reliable gravity inclinometer, was used as the measuring instrument for PROM [35–37]. PROM was measured, also on the normal side, on all visits. PROM was measured in supine lying position for external and internal rotation, and for abduction in standing. The endpoint was when the arm could not be moved more or the pain became unbearable. To avoid discrepancies in measurements due to affection of movements of thumb joints, the distance in Hand-behind-back was measured in centimeters between the styloid process of the radius to the posterior inferior iliac spine. PROM was measured by a research collaborator (a GP) being unaware which group the patients were randomised to. The assessor who took PROM had experience in use of the plurimeter, and had shown acceptable inter-tester reliability [37]. The assessor made entries of the PROM on a separate paper so that confidentiality was maintained from the treating doctor throughout the study.

The time intervals between the consecutive treatments were 1, 1½ and 2 weeks. The control group remained without treatment with corticosteroids in injection or tablet form until 61 days, but could use NSAIDs, Paracetamol or Codeine as needed. SPADI and NPRS were registered on the first visit, after 4 and 8 weeks. The 1 year follow-up for SPADI was only by postal communication.

### Sample size

For SPADI, being the primary outcome measure, we considered an outcome of 20 % better or worse to be clinically significant. This represents a difference in score of 14 at the level of SPADI = 70. Others have considered a difference in score of ≥10 to represent clinically important change [34, 38]. In a previous study where SPADI was a primary outcome measure, the variance in SPADI was 19.8 [27]. Given α = 0.05, we calculated the sample size to be 31 in each group to have an 80 % power to detect a difference in mean SPADI score of ≥14. With a 10 % drop out the number of patients required for the study to have the above mentioned power were calculated to be 34 in each group.

### Statistical analysis

Differences in outcome between the groups were analyzed using repeated measure ANCOVA and regression based ANCOVA. In our analysis we have distinguished between short-term follow-up (4 and 8 weeks) and long-term follow-up (12 months). Since the 4 and 8 weeks data were not independent, we chose to analyze these data as multiple follow-up observations. This was done in a repeated measures ANCOVA model with 4 and 8 weeks observations as repeated measures to capture the main effect of treatment between groups [39] (p.197), and with pretest as a covariate to adjust for baseline differences between subjects. Similarly, we analyzed the long-term follow-up data in another ANCOVA model using a regression procedure with the 12 months observations as dependent variable, group as a categorical independent variable and pretest as a covariate. In an additional/secondary analysis we added other independent variables (specified) to both ANCOVA models to control for possible confounding.

Effect size (ES) for mean change in SPADI was also calculated by subtracting post-test score (8 weeks and 12 months) from baseline in two groups, dividing it by the standard deviation (SD) of the change score:

$$ Effect\  size=\frac{\left[ Mean\  of\  intervention\  group\right]-\left[ Mean\  of\  treatment-as- usual\  group\right]}{Standard\  Deviation} $$

An ES of 0.8 is considered large and of crucial practical or clinical importance, while an ES of 0.2 is considered to be small and without any practical or clinical importance [39].

We performed intention to treat (ITT) analysis [40], keeping patients in their original allocations on randomisation in accordance with ITT principles [41]. We had intervention data for all patients until 8 weeks except for missing data for two patients for 4 weeks and one patient for 8 weeks. One year follow-up data was lacking for six patients. Missing data were imputed following ITT principles.

Software package IBM SPSS Statistics 22 for Windows, was used for all statistical analyses.

**Fig. 1:**
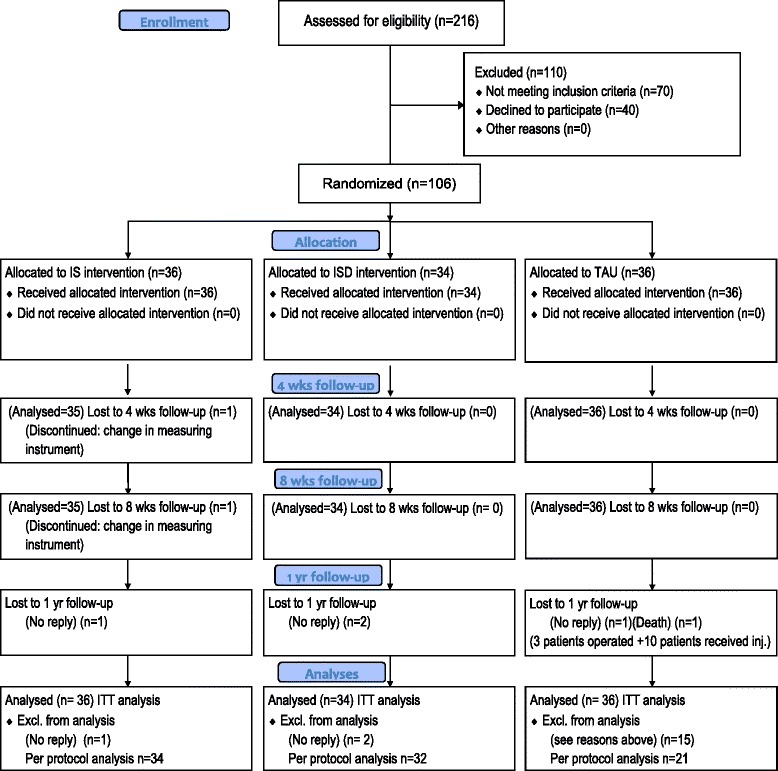
Flow diagram for randomisation and follow-up

We have followed the CONSORT (Consolidated Standards of Reporting Trials) 2010 guidelines for reporting of parallel group randomised trials. Figure 1 included in the manuscript has followed 2010 CONSORT Flow Diagram template. CONSORT 2010 Checklists for Randomised Trials, CONSORT extension for Abstracts Checklist and TIDieR (Template for Intervention Description and Replication) checklist files.

## Results

Of the 216 patients referred for the study, 146 met the inclusion criteria, whereof 40 patients declined to participate for fear of coming in the TAU group and not receiving treatment immediately. Seventy patients were excluded as they were less affected than the specified criteria for reduced ROM or had diabetes. One hundred and six patients were randomised for participation. Thirty-six patients were allocated to the IS group, 34 patients to the ISD group, and 36 patients to TAU (Fig. 1). All completed the specified intervention until 8 weeks, and there were no dropouts, except for one in the IS group. After 1 year 100 patients (95 %) answered the postal questionnaire. One year follow up ended in December 2014. No interim analysis was carried out during the trial.

### Patient characteristics

Baseline characteristics of all the included patients are displayed in Table 1. The three groups were comparable in their baseline regarding age, gender, mean duration of shoulder pain, concurrent neck pain, previously frozen shoulder, number of affected right side and dominant side and sick leaves. There were no statistically significant differences between the three groups regarding side affected, operated shoulder prior to adhesive capsulitis, trauma to shoulder (traumatic adhesive capsulitis), previous shoulder treatment, and smoking. There was a statistically significant difference in use of analgesics at baseline between the two intervention groups (*p* < 0.05), but not between the injection groups and TAU. Furthermore, 11 patients in the distension group had “trauma to shoulder” whereas the IS group had two and the TAU had three patients with previous trauma.

**Table 1:** Baseline characteristics of patients

Characteristics	Injection group Steroid alone (IS)	Injection group Steroid and saline (ISD)	Treatment-as-usual (TAU) group
	Number and % within group *n* = 36	Number and % within group *n* = 34	Number and % within group *n* = 36
Mean age (years)	52 (8.3)	53 (9.2)	54 (6.9)
Female	21 (58 %)	21 (62 %)	19 (53 %)
Duration in months Median (range)	7.5 (2.0–18.0)	7.0 (3.0–37.0)	6.0 (3.0–24.0)
Affected right shoulder	18 (50 %)	12 (35 %)	15 (42 %)
Previous frozen shoulder	6 (17 %)	4 (11 %)	4 (11 %)
Concurrent neck pain	16 (44 %)	15 (44 %)	16 (44 %)
Trauma to shoulder	2 (6 %)	11 (32 %)	3 (8 %)
Previous operation on shoulder	3 (8 %)	3 (9 %)	1 (3 %)
Dominant right side	34 (94 %)	30 (88 %)	34 (94 %)
Previous shoulder treatment	15 (42 %)	22 (65 %)	13 (36 %)
Analgesics	19 (53 %)	14 (41 %)	11 (31 %)
Participants on sick leave	17 (50 %)	16 (47 %)	15 (42 %)
Smokers	8 (22 %)	6 (18 %)	12 (33 %)

### Intervention

Thirty-five patients in the IS group and 34 patients in the ISD group received four injections each within the time frame of 8 weeks. After the intervention period of 8 weeks, 12 patients (33 %) in the TAU group received additional treatment with intra-articular injections with corticosteroid and Lidocaine, same as in the IS group, for pain relief, and three were operated. During the 8 weeks after recruitment, 11 patients in the TAU group had received NSAIDs and/or pain killers as needed, and three patients had received acupuncture for pain relief.

All three groups showed clinically significant change in SPADI from baseline to 8 weeks (>14 points improvement), although both intervention groups had improved significantly more as compared to the TAU group at 8 weeks. Similarly, there was a significant improvement in NPRS at 8 weeks for both intervention groups, but less in the TAU group. Change in PROM for abduction was slightly better between the distension group (54° increased to 69°; i.e. 15° increase) and the TAU group (51° increased to 57°; i.e. 6° increase) at 8 weeks (Table 2).

**Table 2:** SPADI, NPRS and PROM and comparison in outcomes between three groups

	Injection group Steroid alone (IS)	Injection group Steroid and saline (ISD)	Treatment-as-usual (TAU)
	Mean (SD)	Mean (SD)	Mean (SD)
Primary outcome variable			
SPADI			
At inclusion	63.8 (16.0)	60.5 (16.8)	61.9 (19.0)
4 weeks	34.1 (21.4)	30.9 (21.0)	51.9 (22.2)
8 weeks	23.8 (22.0)	20.1 (18.4)	44.4 (23.6)
12 months	16.9 (18.9)	17.2 (19.8)	11.7 (20.3)
Secondary outcome variable			
NPRS			
At inclusion	6.9 (1.4)	7.2 (1.6)	6.6 (2.1)
4 weeks	3.8 (2.2)	3.5 (1.7)	5.6 (2.5)
8 weeks	3.0 (2.3)	2.9 (1.6)	4.7 (2.0)
Tertiary outcome variables			
Abduction (ABD)			
At inclusion	53.7 (13.4)	51.0 (17.8)	50.5 (19.0)
4 weeks	62.7 (15.6)	64.7 (17.2)	53.9 (19.4)
8 weeks	68.9 (15.3)	71.9 (17.0)	56.5 (20.9)
External rotation (ER)			
At inclusion	19.6 (14.7)	25.2 (17.7)	17.3 (13.5)
4 weeks	30.1 (16.3)	35.6 (15.8)	18.8 (14.8)
8 weeks	38.2 (17.6)	42.7 (17.9)	24.0 (18.1)
Internal rotation (IR)			
At inclusion	38.8 (15.5)	41.1 (14.1)	40.2 (15.4)
4 weeks	49.5 (17.4)	52.7 (17.3)	43.7 (16.6)
8 weeks	57.2 (15.7)	59.6 (16.1)	47.3 (18.2)
Hand behind back (HBB)			
At inclusion	0.4 (6.2)	2.2 (7.8)	−0.5 (6.0)
4 weeks	5.9 (7.2)	7.5 (7.8)	1.0 (6.1)
8 weeks	10.1 (6.3)	11.2 (7.2)	4.3 (6.5)

*SPADI* shoulder pain and disability index, *NPRS* numeric pain rating scale, *PROM* passive range of motion

*IS* injection steroid alone, *ISD* injection steroid plus saline, *TAU* treatment-as-usual

**Fig. 2:**
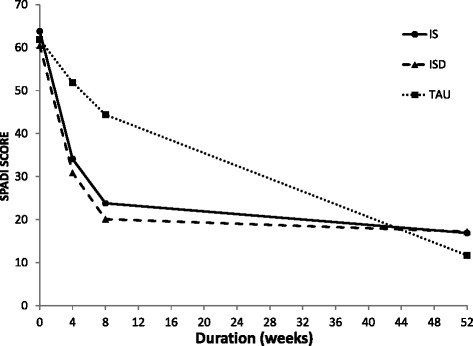
Comparison between intervention and treatment-as-usual groups from inclusion to 52 weeks for SPADI

Both intervention groups had equivalent ES concerning SPADI at 8 weeks (ES 1.2) and 12 months (ES 0.3 and 0.4) (Table 3). At 12 months, however, the change in the TAU group was as large as the change in the two intervention groups and no statistical significant difference was found in SPADI between the three groups, illustrated in Fig. 2.

**Table 3:** Effect size (ES) for SPADI from baseline to 8 weeks and 12 months follow-up for the three groups

SPADI	IS	ISD	TAU	IS & ISD	IS & TAU	ISD & TAU
8 weeks						
Mean change	−40.3	−40.4	−17.4	0.2	22.8	23.0
SD	19.0	19.1	19.8	19.1	19.4	19.4
ES				0.0	1.2	1.2
12 months						
Mean change	−43.0	−39.8	−48.1	3.1	5.1	8.2
SD	19.6	24.7	20.4	22.3	20.0	21.4
ES				0.1	0.3	0.4

*SPADI* shoulder pain and disability index

*IS* injection steroid alone, *ISD* injection steroid plus saline, *TAU* treatment-as-usual

Repeated measure ANCOVA for short-term and regression based ANCOVA for long-term revealed no statistically significant difference between the two intervention groups in SPADI, NPRS and PROM, neither at baseline, nor at short-term, or in SPADI at long-term. A statistically significant change (*p* <0.001) was found for both intervention groups when compared to the TAU group at short-term for SPADI and NPRS. There was a statistically significant difference (*p* < 0.01) at short-term for all PROMs between the two injection groups and TAU (Table 4).

**Table 4:** SPADI, NPRS and PROM: Differences in change scores between the two injection groups (Intervention steroid alone (IS); Intervention steroid plus saline (ISD)) and the treatment-as-usual group (TAU)

	Between groups differences in change, mean (95 % CI)		
	IS vs ISD	IS vs TAU	ISD vs TAU
Primary outcome variable			
SPADI			
Short-term (4 and 8 weeks)^a^	1.2 (−7.1 to 9.6)	−20.8 (−28.9 to −12.7)***	−21.7 (−29.4 to −14.0)***
Long-term (12 months)^b^	0.1 (−10.4 to 10.7)	−7.0 (−16.4 to 2.5)	−7.0 (−16.8 to 2.8)
Secondary outcome variable			
NPRS			
Short-term (4 and 8 weeks)^a^	0.3 (0.6 to 1.2)	−2.0 (−2.8 to −1.1)***	−2.2 (−3.0 to −1.4)***
Tertiary outcome variables			
Abduction			
Short term (4 and 8 weeks)^a^	−4.5 (−9.7 to 0.8)	8.3 (2.3 to 14.3)**	12.7 (6.6 to 18.9)***
External rotation			
Short term (4 and 8 weeks)^a^	−0.9 (−5.8 to 4.1)	10.8 (5.8 to 15.9)***	11.9 (6.8 to 17)***
Internal rotation			
Short term (4 and 8 weeks)^a^	−1.1 (−6.6 to 4.5)	8.8 (3.1 to 14.6)**	9.9 (4.7 to 15.1)***
Hand behind back			
Short term (4 and 8 weeks)^a^	−0.7 (−2.4 to 2.2)	5.0 (2.8 to 7.2)***	5.1 (2.9 to 7.2)***

*SPADI* shoulder pain and disability index, *NPRS* numeric pain rating scale, *PROM* passive range of motion

***p < 0.001, **p < 0.01, *p < 0.05

^a^Repeated measures ANCOVA with baseline value as covariate. Differences and CIs from estimated marginal means

^b^Regression based ANCOVA with baseline value as covariate

In the TAU group, three patients were operated after 8 weeks, and 12 patients chose to receive intra-articular corticosteroid injections without distension. In the intention-to-treat analysis at 12 months, including all patients in the groups to which they were allocated, there were no significant differences between any of the groups regarding change in SPADI (Table 4).

In our study there was only one drop out up to 8 weeks and we did not expect this to affect the results substantially. A secondary per-protocol analysis was performed excluding the 15 patients that did not follow the initial TAU protocol after the 8 week period. This did not affect the results. However, we do acknowledge the fact that exclusion of these patients lowers the sample power for the TAU group.

Five patients (14 %) in the IS group, eight patients (24 %) in ISD group and six patients (14 %) in the TAU group were still on sick leave after 1 year. Eight patients (22 %) in the IS group, nine patients (26 %) in the ISD group and three patients (8 %) in the TAU group were still on medication for shoulder pain at 12 months follow-up.

Six patients (17 %) in the IS group and four (12 %) patients in the ISD group experienced minor transitory side-effects such as flushing and after-pain. No incidences of other side effects were reported. Patients in the two injection groups were asked to guess to which group they belonged to after the last injection. Twenty-six patients (38 %) guessed the wrong group.

## Discussion

Repeated intra-articular steroid injections given with increasing intervals in the gleno-humeral joint gives short-term (8 weeks) benefit. Added capsular distension did not significantly affect the outcome measures for SPADI, NPRS and PROM. However, at long-term follow-up, those who had received no intervention did equally well.

Earlier studies combining distension (10 ml) and corticosteroid versus distension alone and corticosteroid alone, have reported better results for distension [42]. While in studies by Corbeil et al. & Tveitå et al. [30, 31] no significant differences between distension and non–distension arthrography with corticosteroids were found, the main effect might therefore be attributed to corticosteroid alone. Comparing our results between ISD group and TAU group with Tveitå et al. [31], our study has demonstrated larger improvement; for SPADI 24 versus 6, for ABD 15.4 versus 2, for ER 18.7 versus 2 and for IR 12.3 versus 3 respectively. A systematic review concluded with “silver level” evidence for short–term efficacy in pain, ROM, and function of shoulder by arthrographic saline distension and corticosteroid in patients with adhesive capsulitis [28]. Studies with distension and corticosteroid causing capsular rupture performed in hospital settings have also shown significant results [27, 29, 42]. These and other case series studies in primary care with distension and capsular rupture [43, 44] are, however, not comparable to the present study, as capsular rupture was not the intended intervention. We cannot however rule out that capsular rupture might have occurred in some patients. Tveitå et al. [31] have observed capsular rupture at a volume as low as 10 ml.

A dose of 20 mg Triamcinolone was a tradeoff dose between effect and side effects in both intervention groups and is the generally accepted and practiced treatment dose for adhesive capsulitis in primary care. A study by de Jong [45] has shown better effect with a dose of 40 mg Triamcinolone than with 10 mg, whereas another study by Yoon et al. [46] found no significant difference in outcome between a dose of 20 and 40 mg Triamcinolone. In this study we used a series of injections, a total of four over a period of 8 weeks. Many studies with distension have only used a single corticosteroid injection, which makes comparison difficult. Only a few studies have used multiple injections and even fewer have used multiple injections with dilatation [25, 29, 31, 42, 47]. A review has concluded that multiple injections improve pain and ROM in short term from 6 to 16 weeks from the first injection. There is evidence that up to three injections can be beneficial and limited evidence that up to six injections is beneficial [4].

This study has followed the actual practice of treating these patients in primary care with intra-articular injections by landmarks, without fluoroscopic guidance. Some studies with ultrasound guided intra-articular steroid injections claim a short time superiority in pain reduction of about 2 weeks, compared to injections by landmarks [48], which we consider is little as compared to the extra resources required in terms of time and costs.

On 1 year follow-up all three groups had similar outcome, which reflects the natural history of the condition [14, 16, 18, 20, 49]. But the major difference in pain relief (NPRS) and pain and function (SPADI) were recorded in the first 8 weeks in the intervention groups as compared to the control group. From the patient’s perspective, pain relief leading to undisturbed sleep is of great importance [50], which is not so often accredited in studies measuring outcome over time.

One of the strengths of this study is that it is conducted in line with the actual practice in treatment of adhesive shoulder capsulitis in primary care in Norway, i.e. intra-articular steroid injection in gleno-humeral joint by landmarks. There are very few studies that are close to actual practice in treatment of shoulder adhesive capsulitis in primary care [25, 51]. The procedure is safe and simple and easy to learn and cost effective. Only 15 % of patients reported transient side effects and the procedure was not experienced as particularly painful. The limitations of the study are lack of visual verification of delivery of medication in the joint. The injected volume varied from 8 to 20 ml and we cannot assert with certainty that the observed effect was due to distension and not to capsular rupture. Longer time taken in injecting the fluid in the joint might have introduced bias as patients might assume that he or she was in the distension group, which might have been considered the superior method by the patients.

## Conclusion

This intention to treat RCT in primary care indicates that four injections with corticosteroid with or without distension, given with increasing intervals during 8 weeks, were better than treatment-as-usual in adhesive capsulitis of the shoulder. However, in the long run no difference was found between any of the groups, indicating that natural healing takes place independent of treatment.
